# Correlation of Cerebral Microdialysis with Non-Invasive Diffuse Optical Cerebral Hemodynamic Monitoring during Deep Hypothermic Cardiopulmonary Bypass

**DOI:** 10.3390/metabo12080737

**Published:** 2022-08-10

**Authors:** Tiffany S. Ko, Constantine D. Mavroudis, Emilie J. Benson, Rodrigo M. Forti, Richard W. Melchior, Timothy W. Boorady, Vincent C. Morano, Kobina Mensah-Brown, Yuxi Lin, Danielle Aronowitz, Jonathan P. Starr, Tami M. Rosenthal, Brandon C. Shade, Kellie L. Schiavo, Brian R. White, Jennifer M. Lynch, J. William Gaynor, Daniel J. Licht, Arjun G. Yodh, Wesley B. Baker, Todd J. Kilbaugh

**Affiliations:** 1 Department of Anesthesiology and Critical Care Medicine, Children’s Hospital of Philadelphia, Philadelphia, PA 19104, USA; 2 Division of Cardiothoracic Surgery, Cardiac Center, Children’s Hospital of Philadelphia, Philadelphia, PA 19104, USA; 3 Division of Neurology, Department of Pediatrics, Children’s Hospital of Philadelphia, Philadelphia, PA 19104, USA; 4 Department of Physics and Astronomy, University of Pennsylvania, Philadelphia, PA 19104, USA; 5 Division of Perfusion Services, Cardiac Center, Children’s Hospital of Philadelphia, Philadelphia, PA 19104, USA; 6 Geisinger Commonwealth School of Medicine, Scranton, PA 18510, USA; 7 Department of Physics and Astronomy, Johns Hopkins University, Baltimore, MD 21218, USA; 8 Perelman School of Medicine, University of Pennsylvania, Philadelphia, PA 19104, USA; 9 Department of Cardiology, Children’s Hospital of Philadelphia, Philadelphia, PA 19104, USA

**Keywords:** cardiopulmonary bypass, deep hypothermic circulatory arrest, diffuse optics, neuromonitoring, cerebral hemodynamics, cerebral microdialysis, congenital heart surgery, hypoxic-ischemia

## Abstract

Neonates undergoing cardiac surgery involving aortic arch reconstruction are at an increased risk for hypoxic-ischemic brain injury. Deep hypothermia is utilized to help mitigate this risk when periods of circulatory arrest are needed for surgical repair. Here, we investigate correlations between non-invasive optical neuromonitoring of cerebral hemodynamics, which has recently shown promise for the prediction of postoperative white matter injury in this patient population, and invasive cerebral microdialysis biomarkers. We compared cerebral tissue oxygen saturation (StO_2_), relative total hemoglobin concentration (rTHC), and relative cerebral blood flow (rCBF) measured by optics against the microdialysis biomarkers of metabolic stress and injury (lactate–pyruvate ratio (LPR) and glycerol) in neonatal swine models of deep hypothermic cardiopulmonary bypass (DHCPB), selective antegrade cerebral perfusion (SACP), and deep hypothermic circulatory arrest (DHCA). All three optical parameters were negatively correlated with LPR and glycerol in DHCA animals. Elevation of LPR was found to precede the elevation of glycerol by 30–60 min. From these data, thresholds for the detection of hypoxic-ischemia-associated cerebral metabolic distress and neurological injury are suggested. In total, this work provides insight into the timing and mechanisms of neurological injury following hypoxic-ischemia and reports a quantitative relationship between hypoxic-ischemia severity and neurological injury that may inform DHCA management.

## 1. Introduction

Adverse neurodevelopmental outcomes are prevalent in neonates with congenital heart disease who require surgical intervention in the first weeks of life [1,2]. Cyanotic defects have been associated with delayed brain maturation [3], and these neonates have a high risk of post-natal hypoxic-ischemic neurological injury. The inflammatory and metabolic vulnerability of myelinating oligodendrocytes [4] potentially underlies the high incidence (~50%) of white matter injury following cardiac surgery [5,6,7]. Thus, neuromonitoring strategies to identify neurological vulnerability are needed for the detection, treatment, and prevention of neurological injury.

In neonates requiring aortic arch repair, the use of deep hypothermia (DH) in conjunction with cardiopulmonary bypass (CPB) helps mitigate metabolic vulnerability by decreasing the metabolic demands of the brain and thereby permits periods of circulatory arrest that are required to visualize and repair certain cardiac malformations. Deep hypothermic circulatory arrest (DHCA, total cessation of blood flow) and selective antegrade cerebral perfusion (SACP, maintenance of unilateral carotid blood flow via selective cannulation and perfusion of the carotid artery) are the two most commonly employed strategies in neonates undergoing aortic arch surgery. Recently, we reported on the cerebral hemodynamic changes and neurometabolic derangements associated with the use of DHCA and SACP, as well as full-body DHCPB, in a neonatal swine model [8]. The use of non-invasive, continuous diffuse optical measurements of cerebral hemodynamics throughout deep hypothermia permitted subject-specific characterization of hypoxic-ischemic conditions during DHCA, as well as the detection of significant hyperoxic and hypervolemic conditions during full-body DHCPB. Concurrent invasive cerebral microdialysis sampling of the lactate–pyruvate ratio (LPR), a biomarker of metabolic distress, and glycerol, a biomarker of neurological injury, were significantly elevated in both DHCA and DHCPB groups. Looking forward, an optimal level of cerebral oxygenation and perfusion during surgery, that is, neither too low nor too high, is desirable for the prevention of intraoperative neurological injury [9], and non-invasive optical neuromonitoring may be a valuable tool for this task. The lack of knowledge regarding how current management strategies impact the brain has largely limited substantial improvements in postoperative neurological outcomes following congenital heart surgery over the last two decades [10].

To this end, we build upon prior work to examine the association of non-invasive optical metrics of cerebral hemodynamics with invasive neurometabolic biomarkers sampled by cerebral microdialysis. This correlative study was carried out in neonatal swine who were placed on cardiopulmonary bypass and were continuously monitored throughout cooling, deep hypothermia (18 °C), and rewarming to normothermia. We separately examined subjects who underwent circulatory arrest (DHCA) and continuous cerebral perfusion (SACP or DHCPB) to characterize the differential underlying injury mechanisms. In the future, knowledge about these physiologic relationships could inform management strategies based on individual neuromonitoring targets, help optimize DHCPB management, and prevent neurological injury.

## 2. Materials and Methods

In this study, we performed new analyses of data from a prospective, randomized cohort study whose methods have been previously described [8,11]. We examined a subset of thirty neonatal female Yorkshire swine (6–10 days old, 2.5–5 kg) that underwent cardiopulmonary bypass (CPB) support with cooling to deep hypothermia; subjects from the original study that did not undergo cooling were excluded from the present analysis. Invasive sampling of neurometabolic biomarkers of neurological injury is summarized alongside corresponding non-invasive optical measurements of cerebral hemodynamics. The correlation between invasive and non-invasive modalities was assessed to explore the potential utility of cerebral hemodynamic measurements as a quantitative, non-invasive surrogate measure of neurometabolic injury.

### 2.1. Selection of Animal Model

Neonatal swine models have provided foundational data contributions to our understanding of the impact of bypass and circulatory arrest on the brain [11,12,13,14,15,16]. This statement is also true for the clinical translation of diffuse optical techniques for pediatric neuromonitoring [17,18,19,20,21,22,23,24] due to comparability in the anatomical thickness of superficial tissue (i.e., scalp and skull). The animal model used here was developed based on surgical practices at our institution. This investigation builds upon and connects these two bodies of literature to advance the translation of non-invasive optical neuromonitoring to clinical intraoperative management of neonates.

### 2.2. Neurological Monitoring

The methods for neurological monitoring have been previously detailed [8,11,22] but are briefly summarized herein. Following anesthetic induction and intubation, and prior to initiation of cardiopulmonary bypass support, neurological monitoring modalities were placed. These monitoring modalities included nasopharyngeal temperature (NPT, °C) for the guidance of hypothermic therapy, intracranial brain temperature (ICT, °C; CC1-P1, Integra LifeSciences; Plainsboro, NJ, USA), cerebral microdialysis (CMA 71 Elite, mDialysis, Stockholm, Sweden), and non-invasive frequency-domain diffuse optical spectroscopy (FD-DOS) and diffuse correlation spectroscopy (DCS). Invasive measures of ICT and microdialysate samples were acquired in the right frontal cortex, symmetrically contralateral to non-invasive optical neuromonitoring measurements over the left frontal cortex. Following placement, microdialysate samples were collected in 30 min intervals corresponding to experimental periods of normothermic bypass, cooling, deep hypothermia, rewarming, and end of study. Measurements from all other instruments were recorded continuously for the duration of the protocol.

### 2.3. Cardiopulmonary Bypass and Deep Hypothermia

**Figure 1:**
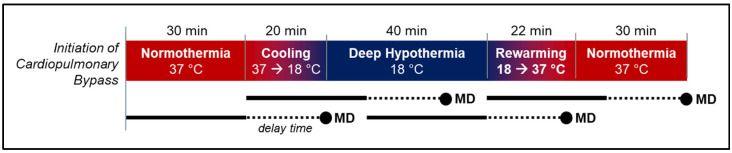
Experimental Protocol and Cerebral Microdialysis (MD) Sampling Timepoints. Following the initiation of cardiopulmonary bypass (CPB) support, animals were maintained at normothermia (nasopharyngeal temperature, NPT, 37 °C) for 30 min. The animals were then cooled to deep hypothermia (NPT 18 °C) and received one of three different perfusion strategies for 40 min: deep hypothermic CPB (DHCPB), deep hypothermic circulatory arrest (DHCA), or selective antegrade cerebral perfusion (SACP). All animals were then returned to CPB support and rewarmed to normothermia. Four MD sampling timepoints corresponding to normothermic bypass, cooling, deep hypothermia, and rewarming are indicated by circles. For each sample, the corresponding 30 min cerebral sampling window is indicated by a solid black line. The dotted lines indicate the 20 min delay time used to account for metabolic reaction time, metabolite “washout” time, and transit time of dialysate from the brain to the MD collection vial (note, the solid and dotted lines for MD samples 2 and 4 are above those for MD samples 1 and 3).

The procedure and timing for the initiation of cardiopulmonary bypass, deep hypothermia, and circulatory arrest or selective antegrade cerebral perfusion are illustrated in Figure 1 and are described in Mavroudis et al. [8,11]. All subjects (n = 30) were stabilized on CPB (flow rate = 150 mL/kg/min) at normothermia (NPT = 37 °C), and baseline measurements were acquired for five minutes. Animals were then cooled to deep hypothermia (NPT = 18 °C) at a target rate of 1 °C per minute. After attainment of 18 °C, the subjects were randomized to receive either continuous, full-body deep hypothermic cardiopulmonary bypass support (DH CPB; n = 10), deep hypothermic selective antegrade cerebral perfusion (SACP; n = 10), or deep hypothermic circulatory arrest (DHCA; n = 10) for 40 min. To initiate DHCA, the CPB arterial outlet flow was turned off and clamped, with venous drainage remaining open for patient exsanguination (i.e., removal of blood from the body). Once the lack of venous return became apparent, the venous drainage was also clamped. Donor blood was added as needed to ensure a minimum hematocrit of 30%. Immediately prior to reinitiating CPB in DHCA animals, 2 meq/kg of sodium bicarbonate was added. Immediately prior to rewarming, 0.5 g/kg mannitol was added in all animals. Following reperfusion of DHCA animals, all groups were rewarmed to normothermia (NPT = 37 °C) at a target rate of 1 °C per minute.

### 2.4. Diffuse Optical Monitoring of Cerebral Hemodynamics

Non-invasive diffuse optical neuromonitoring of cerebral hemodynamics was conducted using the combined techniques of frequency-domain diffuse optical spectroscopy (FD-DOS) and diffuse correlation spectroscopy (DCS). Details regarding FD-DOS/DCS optical instrumentation and data processing have been previously reported [22] and are briefly summarized here.

Using a customized commercial FD-DOS instrument (Imagent, ISS Inc., Champaign, IL, USA) with radio-frequency (110 MHz) intensity-modulated near-infrared light sources and two photomultiplier tube detectors, the AC intensity and phase were measured as a function of source–detector separation. Measurements were acquired at a sampling frequency of 10 Hz from four source–detector separations ranging from 1.5–3 cm. These data were then used to derive absolute tissue absorption and scattering properties ($\mu_{a}$ and ${\mu_{s}}^{\prime }$, respectively) at four wavelengths (λ = 690, 725, 785, and 830 nm) using a semi-infinite approximation [25,26]. Assuming constant cerebral tissue water content of 75% [27], these measured absorption coefficients were then used to quantify cerebral tissue concentrations of oxy- and deoxyhemoglobin ([HbO_2_] and [Hb], respectively; μmol/L) [22,28,29]. Total hemoglobin concentration (THC, μmol/L) and tissue oxygen saturation (StO_2_, %) were computed as:

(1) $$\mathrm{THC}=\;\left[\mathrm{Hb}\right]+\left[{\mathrm{HbO}}_{2}\right]$$

(2) $${\mathrm{StO}}_{2}\left(\%\right)=\frac{\left[{\mathrm{HbO}}_{2}\right]}{\left[\mathrm{Hb}\right]+\left[{\mathrm{HbO}}_{2}\right]}\times 100\%$$

DCS measurements were performed at a source–detector separation of 2.5 cm using custom instrumentation with a continuous-wave, long-coherence length (>10 m), a λ = 785 nm laser source (RCL-080-785S; CrystaLaser Inc., Reno, NV, USA), and two detection arrays of four single-photon-counting avalanche photodiode detectors (SPCM-AQ4C; Excelitas Technologies Corp., Waltham, MA, USA). The DCS calculations of the blood flow index (BFI) incorporated the concurrent measurements of tissue absorption and scattering properties from FD-DOS. BFI was derived by fitting the average (i.e., average across all detectors) hardware-determined (FLEX03OEM-8CH; Correlator.com, Bridgewater, NJ, USA) temporal intensity autocorrelation function using an integration time of 3 s per measurement; for this calculation, we employed solutions to the correlation diffusion equation for a semi-infinite homogenous medium [30].

All continuous time-series data were synchronized using 15 s epoch averages. Measures of relative THC and cerebral blood flow (rCBF; %) at timepoint t were computed as:

(3) $$\mathrm{rTHC}\left(t\right)=\frac{\mathrm{THC}\left(t\right)}{{\mathrm{THC}}_{\mathrm{baseline}}}\times 100\%$$

(4) $$\mathrm{rCBF}\left(t\right)=\frac{\mathrm{BFI}\left(t\right)}{{\mathrm{BFI}}_{\mathrm{baseline}}}\times 100\%$$

Here, the baseline THC and BFI values were calculated as the mean THC and BFI measured during a 5 min baseline period immediately prior to cooling.

### 2.5. Cerebral Microdialysis

Cerebral microdialysis is an established, minimally invasive, clinical neurometabolic monitoring technique that enables the direct sampling of metabolite concentrations in the interstitial fluid of the brain [31]. A microdialysis sampling catheter (10 mm membrane length, 60 mm shaft length, and 220 mm outlet tubing length; 70 Brain Microdialysis Catheter 60/10, mDialysis, Stockholm, Sweden) was primed with sterile saline, inserted 1–1.5 cm deep into the parenchyma of the brain, and dialysate was continuously acquired at a rate of 1 µL/min. Following a minimum equilibration period of 30 min, dialysate samples were collected in 30 min intervals. The duration of the equilibration period is in line with current clinical practice [31,32] and provided time for (1) the diffusion of cerebral metabolites into the prime fluid and the transit of this equilibrated dialysate through the length of the outlet tubing to the collection vial and (2) recovery of the blood–brain barrier following membrane insertion. Collected dialysate samples were analyzed for concentrations of pyruvate, lactate, glycerol, and glucose (ISCUS FlexTM Microdialysis Analyzer, mDialysis, Stockholm, Sweden). These metabolite concentrations reflect the balance of metabolic substrate delivery and metabolism and are thus highly sensitive biomarkers of hypoxic-ischemic derangements resulting in “energy failure”. Specifically, elevation of the lactate–pyruvate ratio (LPR) occurs in the setting of anaerobic respiration and low oxygen availability [31]. While typically <25 under normal conditions, the persistence of insufficient oxygen delivery during hypoxic-ischemia leads to increasing lactate production and the elevation of LPR; LPR >40 is a generally accepted threshold defining cerebral metabolic distress/crisis and has been associated with the degree of brain atrophy and functional outcomes in traumatic brain injury (TBI) patients [31,33,34].

Elevated glycerol has also been repeatedly associated with mortality and poor neurological outcomes in clinical TBI patients [35]. Glycerol concentrations in the brain are known to accumulate via uptake of systemically circulating glycerol, glycolytic breakdown of glucose, or breakdown of glycerophospholipids (also known as phosphoglycerides) [32,36]. In the setting of neurological injury, the latter mechanism predominates due to the degradation of glycerophospholipids in cell membranes during apoptosis [37,38]. Normal cerebral glycerol concentrations have been observed to range between 50–100 µmol/L in humans [39]; in TBI patients, glycerol concentrations exceeding 80–150 µmol/L have been associated with poor outcomes, including increased mortality [35].

Thus, the lactate–pyruvate ratio (LPR) and glycerol concentrations were incorporated into the present analysis as surrogate measures of metabolic distress and neurological injury, respectively [31,36].

### 2.6. Statistical Analysis

All statistical analyses were carried out using MATLAB 2020a (The MathWorks Inc., Natick, MA, USA).

#### 2.6.1. Longitudinal Changes in Cerebral Physiology

For each microdialysis sample, corresponding values of non-invasive, optically measured cerebral hemodynamic parameters and invasive intracranial temperature were extracted from continuous monitoring data. Optically measured cerebral hemodynamic parameters included the cerebral tissue blood oxygen saturation (StO_2_, %), relative total hemoglobin concentration (rTHC, % baseline), and relative cerebral blood flow (rCBF, % baseline). Based on a delay time sensitivity analysis (see Supplementary Materials), a 20 min delay time between microdialysis sampling and cerebral hemodynamic parameters was incorporated to account for the metabolite reaction time, the “washout” time for the metabolite to enter the extracellular space, and the transit time of the dialysate from the brain to the collection tube. Thus, corresponding cerebral hemodynamic parameter values were calculated as the mean of 30 min of continuous data, which concluded 20 min prior to the time of dialysate collection (Figure 1).

For each experimental period, differences in physiologic parameters between groups (DHCPB, SACP, or DHCA) were examined using the non-parametric Kruskal–Wallis test followed by post hoc pairwise comparisons. A Bonferroni correction for multiple comparisons was employed to identify significant pairwise group differences using an adjusted significance level of *p* < 0.05.

#### 2.6.2. Correlation between Cerebral Hemodynamics and Biomarkers of Neurological Injury

Linear mixed-effects models with random slope and intercept effects were applied to examine the relationship between microdialysis biomarkers of neurological injury (LPR, glycerol) and cerebral hemodynamic parameters. In our prior study, cerebral hemodynamics and microdialysis biomarkers during deep hypothermia were found to be comparable between DHCPB and SACP groups, with the exception of DHCPB exhibiting modestly higher StO_2_ compared to SACP (median [IQR] = 73.5% [65.6, 75.4] vs. 64.4% [54.7, 67.7], *p* = 0.033) [8]. In contrast, DHCA was found to have significantly lower StO_2_, rTHC, and rCBF and higher LPR and glycerol concentrations compared to all other groups. Thus, relationships were separately assessed for animals that received continuous deep hypothermic cerebral perfusion (DHCPB and SACP groups, “No DHCA”) versus animals that underwent circulatory arrest (DHCA). The normality of parameters was assessed using the Kolmogorov–Smirnov test [40]. Both LPR and glycerol concentration were found to have significant non-normality and exhibited positively skewed distributions. Thus, microdialysis biomarkers were log-transformed prior to correlation analysis. The significance of correlations was assessed using the *p*-value of the fitted slope. The goodness of fit to a linear relationship was evaluated by adjusted R^2^.

Initial correlation analysis was performed using a 20 min delay time between cerebral hemodynamics and microdialysis sampling. Secondary correlation analysis was also performed using a 60 min delay time to examine the effect of delay time on the relationship between microdialysis parameters and cerebral hemodynamics. Cerebral hemodynamic values were similarly calculated as the mean of 30 min of continuous data, which concluded 60 min prior to the time of dialysate collection.

#### 2.6.3. Non-Invasive Predictors of Cerebral Metabolic Distress and Injury

After detecting significant correlations between microdialysis and cerebral hemodynamic parameters in DHCA animals, a post hoc analysis was performed to suggest thresholds for individual parameters that may indicate neurologic vulnerability during or following DHCA. Neurologic vulnerability was indicated by either cerebral metabolic distress, defined as an LPR >40 using a 20 min sampling delay, or neurological injury, defined as a glycerol concentration >100 µmol/L using a 60 min sampling delay. For this analysis, measured glycerol concentrations were adjusted to account for <100% recovery of the true tissue glycerol concentration. The recovery rate is significantly influenced by the flow rate and catheter length; faster flow rates and smaller membranes reduce recovery. Based on the use of a 10 mm microdialysis membrane and 1 µL/min flow rate in this study, an expected recovery rate of 30% has been reported [41]. An unadjusted sampling threshold of >30 µmol/L would approximate a true tissue glycerol concentration threshold of >100 µmol/L after adjusting for the recovery rate.

Odds ratios and suggested metabolic distress and neurologic injury thresholds for StO_2_, relative THC, and relative CBF were determined based on univariate logistic regression models. Parameter threshold values corresponded to a predicted probability of 0.5 for the binary outcomes of LPR >40 or adjusted glycerol >100 µmol/L, respectively.

## 3. Results

### 3.1. Summary of Experimental Characteristics

Successful diffuse optical monitoring and cerebral microdialysis (MD) sampling was achieved in 7 of 10 DHCPB animals, 7 of 10 DHCA animals, and 6 of 10 SACP animals; the fallout of the 10 remaining animals was due to incidental obstruction of the optical signal by cutaneous bleeding (n = 9/10) or malfunction of the MD pump (n = 1/10).

### 3.2. Longitudinal Changes in Cerebral Physiology

**Figure 2:**
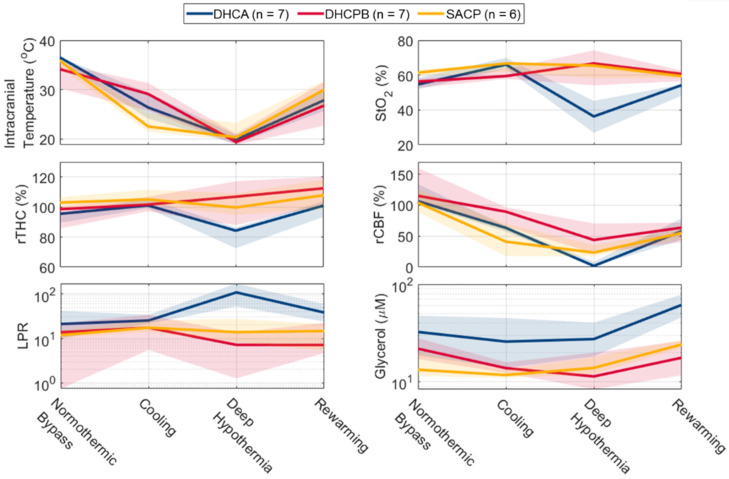
Summary of Cerebral Physiologic Parameters at Microdialysis Sampling Timepoints. The median (*bold line*) and interquartile range (*shaded*) within each group are summarized for measured physiologic parameters at each microdialysis sampling timepoint. *Abbreviations*: DHCA, deep hypothermic circulatory arrest; DHCPB, deep hypothermic cardiopulmonary bypass; SACP, selective antegrade cerebral perfusion; rTHC, relative total hemoglobin concentration; LPR, lactate–pyruvate ratio; StO_2_, tissue oxygen saturation; rCBF, relative cerebral blood flow.

Microdialysis biomarkers and corresponding physiologic parameters are summarized by group and plotted as a function of the experimental period in Figure 2. Samples corresponding to the post-rewarming normothermic bypass timepoint were not consistently collected in all subjects and were excluded from the timepoint-specific analysis (therefore, this timepoint is not shown in Figure 1).

As anticipated, corresponding intracranial temperature values approach deep hypothermia in all animal groups and recover towards normothermia during rewarming. During initial normothermic bypass, significant differences were observed in DHCA animals for glycerol concentrations and the corresponding intracranial temperature (ICT) and relative THC values (Table 1). DHCA animals exhibited a small elevation in ICT compared to DHCPB animals (median = 36.5 vs. 34.2 °C) but not SACP animals; DHCA animals exhibited moderately lower relative THC compared to SACP (95.5 vs. 102.9%); and DHCA animals exhibited elevated glycerol concentration compared to SACP (32.3 vs. 13.3 µmol/L). In samples collected during cooling, the elevation of glycerol in DHCA animals compared to SACP persisted (25.8 vs. 11.7 µmol/L); no other significant differences were detected. Variability in the duration and amount of anesthetic exposure and its impact on tissue metabolism could potentially have resulted in variable concentrations of glycerol in the brain prior to randomization.

The impact of differing perfusion strategies became evident in cerebral hemodynamics during deep hypothermia. No significant differences were observed in intracranial temperature, suggesting uniform effects of temperature on microdialysis metabolite sampling across groups. Significant differences in the DHCA group were found in StO_2_, relative THC, relative CBF, and LPR. The DHCA group exhibited significantly lower StO_2_ versus both DHCPB and SACP groups; lower relative THC versus both DHCPB and SACP groups; lower relative CBF versus both DHCPB and SACP groups; and higher LPR versus the DHCPB group. These group differences demonstrate significantly greater severity of hypoxic-ischemia in DHCA animals, reflected by both quantitative measurements of oxygenation and perfusion as well as invasively sampled markers of metabolic distress (elevated LPR).

During rewarming, significantly lower StO_2_ persisted in the DHCA group (median = 54.3%) versus both DHCPB (60.7%) and SACP (59.4%) groups. The DHCA group also demonstrated significantly elevated glycerol concentration versus the DHCPB group (61.3 vs. 17.6 µmol/L). No significant differences were detected in other physiologic parameters at this time point, indicating the resolution of metabolic distress. Thus, following return to bypass support, DHCA animals demonstrated recovery of cerebral perfusion that was comparable to other perfusion strategies that did not entail circulatory arrest. The presence of elevated glycerol during rewarming but not during deep hypothermia in DHCA animals suggests a contributing role of ischemia–reperfusion injury or a delay in either the generation of glycerol or its “washout” into sampled interstitial fluid following hypoxic-ischemic injury.

No significant differences were observed between DHCPB and SACP groups in any of the measured parameters at any timepoint. The distinct time course of cerebral hemodynamics and injury expression observed in the DHCA group versus DHCPB and SACP groups motivated the distinct examination of quantitative relationships.

### 3.3. Correlation between Cerebral Hemodynamics and Biomarkers of Neurological Injury

#### 3.3.1. Lactate–Pyruvate Ratio (LPR)

**Figure 3:**
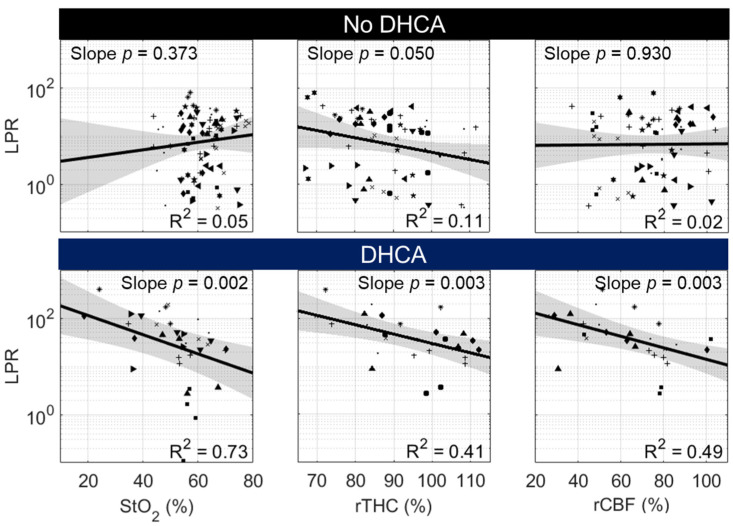
Correlation between the Cerebral Lactate–Pyruvate Ratio (LPR) and Cerebral Hemodynamics in “No DHCA” and DHCA Groups. Correlation data from individual animals are designated by a unique symbol within “No DHCA” animals (i.e., animals randomized to DHCPB and SACP groups) and, separately, within DHCA animals. No significant slope effect was detected in linear mixed-effects models examining the relationship between log-normalized LPR and StO_2_, rTHC, or rCBF data acquired during and following deep hypothermia in the “No DHCA” animals. By contrast, DHCA animals demonstrated a significant negative slope effect (*p* < 0.05) in identical models.

Here, we explore potential correlations between non-invasive measures of oxygenation and perfusion and LPR to determine if these non-invasive measures may be used in a quantitative manner to assess the severity of metabolic distress under operative conditions. Fitted linear mixed-effects models examining the relationship between LPR and corresponding cerebral hemodynamic parameters, separately assessed for animals that received continuous deep hypothermic cerebral perfusion (DHCPB and SACP groups, “No DHCA”) versus animals that underwent circulatory arrest (DHCA), are shown in Figure 3. To highlight the physiologic relationship of different perfusion strategies, only data acquired during and following deep hypothermia (i.e., following group randomization) were included in the analysis.

In animals that received continuous perfusion to the brain (DHCPB and SACP), neither StO_2_ nor rCBF was correlated with LPR. A significant inverse correlation was observed between rTHC and LPR, indicating that increased blood volume may facilitate oxygen delivery to tissue in the setting of adequate oxygen delivery. In animals that underwent DHCA, LPR was significantly inversely correlated with all cerebral hemodynamic parameters. Reduced cerebral oxygenation, blood volume, and cerebral blood flow were all found to increase LPR. Notably, the linear model fit between StO_2_ and log-normalized LPR demonstrated strong linearity (R^2^ = 0.73).

#### 3.3.2. Glycerol

**Figure 4:**
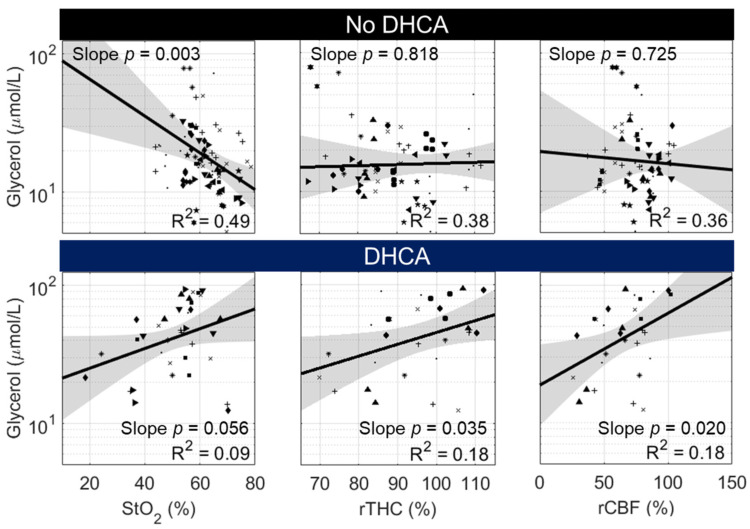
Correlation between Cerebral Glycerol and Cerebral Hemodynamics in “No DHCA” and DHCA Groups. Correlation data from individual animals are designated by a unique symbol within “No DHCA” animals (i.e., animals randomized to DHCPB and SACP groups) and, separately, within DHCA animals. In “No DHCA” animals, a significant negative slope effect was detected in the linear mixed-effects model examining the relationship between log-normalized glycerol concentration and StO_2_, but not with rTHC or rCBF. Examining identical models in DHCA animals, trending (*p* < 0.01) or significantly (*p* < 0.05) positive slope effects were observed between log-normalized glycerol and all three parameters.

In the setting of neurological injury, elevated glycerol is observed secondary to the degradation of cell membranes during cell death [37]. Here, we examine the correlation of non-invasive cerebral hemodynamic parameters with glycerol to determine if diagnostic injury information could potentially be accessed non-invasively. Fitted linear mixed-effects models examining the relationship between glycerol concentration and corresponding cerebral hemodynamic parameters are shown in Figure 4.

In animals that received continuous deep hypothermic cerebral perfusion (DHCPB and SACP), glycerol was significantly inversely correlated with StO_2_ (*p* = 0.003), but glycerol did not demonstrate a significant correlation with relative THC or relative CBF. Secondary analysis (not shown) of the relationship between the change in glycerol from initial normothermic bypass versus StO_2_ also demonstrated a highly significant correlation. The majority of data points reflected a decrease in glycerol concentration from initial normothermic bypass. Thus, the observed inverse correlation between glycerol and StO_2_ should be interpreted in the setting of adequate oxygen delivery; in this case, reductions in glycerol are potentially associated with reduced cerebral oxygen metabolism and not necessarily a reduction in neurological injury.

In DHCA animals, glycerol was weakly correlated with rTHC and rCBF (0.01 < *p* < 0.05) and trended with StO_2_ (*p* = 0.056). This relationship was in opposition to our hypothesis that glycerol, as a surrogate measure of neurological injury, would be elevated following transient hypoxia (low StO_2_) and ischemia (low rTHC and low rCBF). However, all models demonstrated poor linear fits (R^2^ < 0.2). These poor fits may be a consequence, in part, of a temporal delay between the presence of hypoxic-ischemic conditions and the related elevation in glycerol.

**Figure 5:**
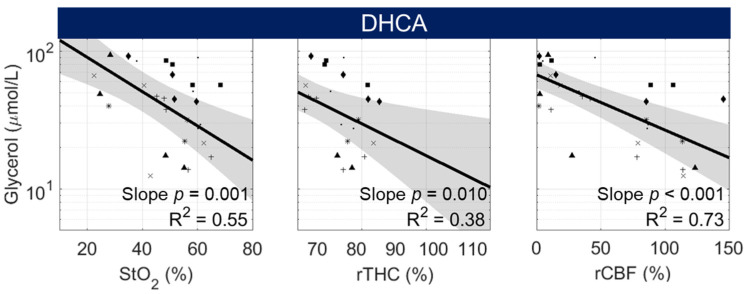
Correlation between Cerebral Glycerol and Cerebral Hemodynamics in DHCA Animals with a 60 min Delay between Sampling Methods. Correlation data from individual animals are designated by a unique symbol within DHCA animals (i.e., animals randomized to DHCPB and SACP groups). When accounting for a 60 min delay between cerebral hemodynamics and microdialysis sample collection from which glycerol concentrations were analyzed, in DHCA animals, a significant negative slope effect was detected in the linear mixed-effects model examining the relationship between log-normalized glycerol concentration and StO_2_, rTHC, and rCBF. No significant correlations were observed at this delay time in the “No DHCA” animals.

Thus, we also looked at the correlation between glycerol and cerebral hemodynamics using a 60 min delay time. The improved goodness of fit observed in our delay-time sensitivity analysis further supported this additional analysis (see Supplementary Materials). In contrast to models fit using a 20 min delay time, we observed significant negative correlations between glycerol and StO_2_ (*p* = 0.001; R^2^ = 0.55), rTHC (*p* = 0.01, R^2^ = 0.38), and rCBF (*p* < 0.001, R^2^ = 0.73; Figure 5). These results suggest that in DHCA animals, hypoxic-ischemia-related elevation of glycerol is temporally delayed and follows the elevation of LPR. In animals that did not undergo DHCA, no significant correlations were observed between glycerol and cerebral hemodynamics at this delay time.

### 3.4. Non-Invasive Predictors of Cerebral Metabolic Distress and Injury

Following the demonstration of significant correlations between microdialysis and cerebral hemodynamic parameters in DHCA animals, a post hoc analysis was performed to suggest thresholds for individual parameters that may indicate cerebral metabolic distress (LPR >40) [33] or neurological injury (glycerol >100 µmol/L) [39]. The odds ratio for each parameter and suggested prediction thresholds are listed in Table 2 and Table 3. Suggested thresholds for StO_2_ and rTHC are similar between outcomes; a lower rCBF threshold is suggested for glycerol versus LPR-based outcomes (9.4% vs. 39.0%).

## 4. Discussion

Non-invasive optical neuromonitoring of cerebral hemodynamics is a promising tool to detect intraoperative cerebral metabolic distress during deep hypothermic cardiopulmonary bypass (DHCPB) and circulatory arrest (DHCA). In a high-fidelity large animal model of DHCA, cerebral tissue oxygen saturation (StO_2_), relative blood volume, and relative CBF were all negatively correlated with invasive cerebral microdialysis (MD) sampling of the lactate–pyruvate ratio (LPR) and glycerol concentration. However, hypoxic-ischemic elevation of LPR occurred more acutely (20 min delay time) than the elevation of glycerol (60 min delay time). Individual cerebral hemodynamic parameter thresholds that may indicate cerebral metabolic distress and neurological injury are derived and reported. These thresholds provide preliminary guidance for the interpretation of cerebral hemodynamic parameters, aiming to detect neurological vulnerability, as well as intraoperative management guidance to mitigate hypoxic-ischemic injury.

Hypoxic-ischemic injury is known to cause cerebral metabolic distress due to inadequate delivery of metabolic substrates for energy production [42]. Reduced oxygen availability leads to increased anaerobic metabolism of pyruvate into lactate versus aerobic metabolism of pyruvate via oxidative phosphorylation. Thus, elevated LPR is consistently observed in the setting of hypoxic-ischemia [31] and is a specific biomarker of reduced oxygen delivery. Following persistent depletion of metabolic substrates, breakdown of ATP-dependent, homeostasis-maintaining membrane ion channels results in a cytotoxic increase in intracellular calcium (Ca^2+^) [42]. This, in turn, activates phospholipases and proteases that degrade membrane glycerophospholipids, leading to apoptosis/cell death. Glycerophospholipids are the predominant component of mammalian cell membranes [43] and are estimated to make up 4–5% of the total wet weight of the brain [44]. These characteristics likely underlie the utility of glycerol as a quantitative biomarker of neurological injury severity. Elevated cerebral LPR and glycerol have been observed in animal models of hypothermic CPB with SACP and DHCA [8,11,45,46,47,48]. In our work, we further examine these types of quantitative relationships with clinically translatable, non-invasive optical monitoring metrics of cerebral hemodynamics.

Initially, we selected a delay time of 20 min between measured cerebral hemodynamic parameters and microdialysis sample collection. A minimum delay time of 5 min is expected based on our microdialysis sampling characteristics [49]. However, this does not account for chemical reaction times leading to the production of measured metabolites or delays in their extracellular expression, via either active or passive transport, and diffusion to the sampling catheter (i.e., “washout” time) that enables detection by microdialysis. The rate of diffusion in the extracellular space is dependent on several biomechanical and electrochemical properties of the brain that have been shown to be significantly impacted by hypoxic-ischemia [50]. Due to the lack of sensitivity to the intracellular environment, we were unable to differentially characterize contributions to this delay using monitoring modalities in the present study. Thus, we empirically selected a 20 min delay time based on evaluating the optimal significance of the correlation and goodness of fit of linear models (see Supplementary Materials). Using this delay time, we detected the expected inverse relationship between LPR and all cerebral hemodynamics in DHCA animals. We did not observe a correlation in non-DHCA animals for measures of oxygenation (StO_2_) or perfusion (rCBF). Note that, among non-DHCA animals, only 4/68 evaluable samples exhibited metabolic distress (LPR >40); this lack of injury likely precluded the detection of the expected association. Given that LPR is highly sensitive to deficits in oxygen delivery, this provides evidence that non-invasive optical neuromonitoring is able to quantitatively assess the severity of hypoxic-ischemia during DHCA. Application of the suggested parameter thresholds in the intraoperative setting could assist in the clinical optimization of DHCA management by providing real-time assessment of hypoxic-ischemic exposure. Clinical interventions, including temperature management and intermittent perfusion, could be used to either decrease metabolic demand or briefly replenish oxygen content.

We also examined the relationship between glycerol and cerebral hemodynamics using a 20 min delay time. With this delay, we uncovered an inverse relationship between glycerol and StO_2_, but not rTHC or rCBF, in animals with continuous deep hypothermic support. Glycerol levels were relatively normal in these animals, thereby suggesting that the observed relationship may simply reflect intrinsic fluctuations in glycerol concentration associated with glycolytic metabolism [39]. In DHCA animals, we observed a positive correlation between glycerol concentration and all cerebral hemodynamic parameters. This relationship was unexpected; we had hypothesized that decreased oxygenation and perfusion that lead to hypoxic-ischemic injury would be negatively correlated with neurological injury, as reflected by the elevation of glycerol. Thus, we suspected that these initial results were a function of the timing of glycerol sampling relative to cerebral hemodynamic measurements of hypoxic-ischemic injury. While glycerol elevation following transient ischemic injury in mice has been detected 15–30 min after injury, it continues to rise until stabilization after 1–2 h [51]. In contrast, LPR was observed to rise within 15 min, peak within 30min post-injury, and return to baseline by 60 min. A 30 min offset between the peak of LPR and glycerol was also observed in our dataset and in prior swine models of DHCA [47,48].

To further explore the delay time between LPR and glycerol in our data, we performed a sensitivity analysis examining the correlation between LPR and glycerol in DHCA animals at different delay times. Notably, no correlation was observed when LPR and glycerol were compared within the same microdialysis sample (no delay time), but a correlation was observed using a 30 or 60 min delay between LPR and glycerol values (see Supplementary Materials). To explore this issue more deeply, we conducted an additional analysis examining the correlation between glycerol and cerebral hemodynamics using a longer delay time of 60min between cerebral hemodynamics and the microdialysis sampling time. For the 60 min delay time, we observed the hypothesized negative correlation between glycerol and all cerebral hemodynamic parameters. In light of this observation, the presence of a positive correlation of oxygenation and perfusion with glycerol at 20 min may reflect the additional contribution of secondary ischemia–reperfusion injury [52] following primary hypoxic-ischemic insult. This secondary injury is thought to arise from the generation of excessive reactive oxygen species during reperfusion due to dysregulation of the mitochondrial respiratory chain and loss of mitochondrial membrane integrity [38]. Taken together, the results and additional temporal analysis provide valuable insights into the timing and mechanisms of neurological injury following hypoxic-ischemic injury. There is evidence of a significant time delay (30–60 min) between hypoxic-ischemic insult and neurological injury; however, injury associated with ischemia–reperfusion may present more acutely (<20 min). Thus, non-invasive quantification of cerebral hemodynamics may also provide valuable diagnostic information regarding the management of reperfusion following DHCA. Further examination of the impact of modulating the bypass flow rate and oxygen content during reperfusion on neurological outcomes is warranted.

### Limitations

This study has several limitations. Intracranial pressure (ICP) is an important variable that was not explicitly measured in this study and may impact measured cerebral hemodynamics. For example, during rapid rewarming, ICP could increase, resulting in a decrease in cerebral perfusion pressure and cerebral blood flow. Future work should consider the explicit measurement of ICP.

The timing and recovery of extracellular concentrations of metabolites by microdialysis are known to vary based on sampling characteristics and catheter placement within the tissue. For the 10 mm catheter and 1 µL/min infusion rate used, a 30% solute concentration recovery is anticipated [41]. A correction factor was used to approximate the true glycerol concentration for the estimation of cerebral hemodynamic thresholds. This correction and other physiologic factors that impact baseline concentrations and recovery should be taken into consideration when comparing our reported values to the literature. The use of similar operative anesthesia in humans in Reinstrup et al., 2000 (fentanyl, isoflurane, and propofol; used in this study: fentanyl, isoflurane, and dexmedetomidine) resulted in reduced glycerol concentrations (mean [SD] = 28 [16] µmol/L) compared to samples acquired when awake (42 [29] µmol/L) but did not affect LPR (22 [6] vs. 21 [6]) [39]. Notably, these values were acquired using identical microdialysis sampling characteristics and resulted in comparable values to the baseline values reported in the present neonatal swine cohort.

Regional variability in brain metabolism is well-documented and is another potential source of uncertainty in our results; the cortex consumes more than twice as much glucose as white matter [53]. In the immature brain, myelinating oligodendrocytes in white matter are known to be particularly vulnerable to inflammation and hypoxic-ischemic insults [4]. In this study, precise catheter location was not confirmed by imaging following insertion but was consistently placed at a fixed depth into the brain parenchyma. Variability in catheter location may have influenced baseline concentrations and the relationship between metabolic distress and cellular injury based on the relative contribution of the cortex and white matter to the sampled interstitial fluid. Furthermore, it was not possible to perform invasive microdialysis sampling co-localized with non-invasive optical neuromonitoring. Thus, additional variability in our data is introduced by the assumption that both modalities are separately sampling the same global changes within the brain. Future studies with multiple (e.g., left and right hemispheres, frontal and occipital) optical and microdialysis sampling locations are necessary to verify these assumptions.

## Figures and Tables

**Table 1:** Comparison of Cerebral Physiologic Parameters at Microdialysis Sampling Timepoints.

Experimental Timepoint	Parameter	DHCA (n = 7)	DHCBP (n = 7)	SCP (n = 6)
Normothermic Bypass	ICT, °C	36.5 [35.6, 36.6] *	34.2 [30.3, 35.2] *	35.8 [34.6, 36.4]
	StO_2_, %	54.7 [51.9, 58.4]	56.3 [52.7, 57.7]	61.4 [59.5, 63.2]
	rTHC, % Baseline	95.5 [89.5, 96.1] ^†^	98.5 [85.7, 100.5]	102.9 [99.2, 106.5] ^†^
	rBFI, % Baseline	106.6 [102.8, 133.9]	115.2 [105.0, 160.1]	104.1 [88.8, 125.1]
	LPR	21.2 [10.0, 41.8]	13.8 [0.7, 22.0]	11.9 [10.3, 15.3]
	Glycerol, µmol/L	32.3 [18.6, 47.6] ^†^	21.8 [17.1, 27.9]	13.3 [11.4, 20.3] ^†^
Cooling	ICT, °C	26.4 [24.2, 29.4]	29.2 [25.8, 31.4]	22.6 [21.5, 25.3]
	StO_2_, %	66.1 [63.7, 69.9]	59.5 [57.8, 63.6]	66.8 [61.8, 66.8]
	rTHC, % Baseline	101.1 [99.1, 105.5]	101.6 [97.2, 106.7]	105.1 [101.5, 111.5]
	rBFI, % Baseline	63.3 [57.2, 66.2]	89.5 [65.6, 96.6]	41.0 [17.9, 70.3]
	LPR	25.3 [21.5, 34.1]	17.5 [5.5, 34.2]	17.5 [14.0, 25.4]
	Glycerol, µmol/L	25.8 [13.8, 45.0] ^†^	13.8 [12.3, 15.8]	11.7 [10.9, 13.3] ^†^
Deep Hypothermia	ICT, °C	19.9 [19.0, 20.8]	19.4 [18.9, 21.1]	20.4 [19.7, 23.3]
	StO_2_, %	36.3 [26.9, 45.2] *^,†^	66.7 [54.2, 74.3] *	65.5 [58.2, 67.0] ^†^
	rTHC, % Baseline	84.2 [72.6, 86.3] *^,†^	106.8 [87.9, 117.1] *	99.7 [94.7, 109.0] ^†^
	rBFI, % Baseline	1.8 [1.3, 8.2] *^,†^	43.8 [22.6, 70.3] *	23.4 [17.3, 35.4] ^†^
	LPR	108.9 [52.7, 172.3] *	7.2 [1.3, 13.5] *	13.9 [8.2, 27.7]
	Glycerol, µmol/L	27.4 [18.2, 40.2]	11.3 [8.3, 19.9]	13.8 [11.9, 18.0]
Rewarming	ICT, °C	27.9 [25.7, 29.8]	26.8 [22.8, 31.6]	30.0 [27.0, 31.8]
	StO_2_, %	54.3 [48.3, 54.6] *^,†^	60.7 [56.5, 62.6] *	59.4 [58.3, 61.8] ^†^
	rTHC, % Baseline	101.0 [93.5, 103.6]	112.5 [101.4, 120.2]	107.8 [98.8, 118.3]
	rBFI, % Baseline	58.0 [43.9, 78.8]	63.8 [40.3, 71.9]	55.2 [45.8, 61.3]
	LPR	38.3 [24.0, 59.1]	7.1 [4.7, 22.6]	14.7 [5.2, 21.1]
	Glycerol, µmol/L	61.3 [45.8, 78.4] *	17.6 [11.6, 26.2] *	24.1 [22.3, 26.9]

Symbols denote significant differences between DHCA and DHCPB groups (*) or DHCA and SCP groups (^†^) determined by adjusted *p*-value < 0.05 following Bonferroni correction.

**Table 2:** Odds Ratios and Thresholds for Cerebral Hemodynamic Prediction of LPR >40.

Parameter	Odds Ratio [95% CI]	*p*-Value	P(x) = 0.5 Threshold
StO_2_ (%)	0.86 [0.76, 0.97]	0.010	48.2
rTHC (% Baseline)	0.89 [0.81, 0.98]	0.010	91.0
rCBF (% Baseline)	0.94 [0.90, 0.98]	0.003	39.0

**Table 3:** Odds Ratios and Thresholds for Cerebral Hemodynamic Prediction of Glycerol >100 µmol.

Parameter	Odds Ratio [95% CI]	*p*-Value	P(x) = 0.5 Threshold
StO_2_ (%)	0.91 [0.86, 0.96]	<0.001	47.8
rTHC (% Baseline)	0.90 [0.85, 0.95]	<0.001	88.0
rCBF (% Baseline)	0.98 [0.97, 1.00]	0.007	9.4

## Data Availability

The raw data used for analysis in this study are reported within figures in the manuscript text and in Supplementary Materials. Additional data formats are available on request from the corresponding author.

## Supplementary Materials

Supporting information can be downloaded at: https://www.mdpi.com/article/10.3390/metabo12080737/s1.
