# Supplementary material for: Correlation of Cerebral Microdialysis with Non-Invasive Diffuse Optical Cerebral Hemodynamic Monitoring during Deep Hypothermic Cardiopulmonary Bypass
Source: Metabolites. 2022 Aug 10;12(8):737. doi: 10.3390/metabo12080737 (PMC9416552; doi:10.3390/metabo12080737)
Supplement: Supplementary file 1 [file metabolites-12-00737-s001.zip › metabolites-1853095-supplementary.pdf]

Supplementary Materials

Sensitivity Analysis of Delay Time between Cerebral Microdialysis Sample Collection and Cerebral Hemodynamics

We examined the correlation of cerebral microdialysis (MD) metabolite values and cerebral hemodynamics using a range of delay times (10 to 60 minutes in 5-minute steps) between time of microdialysate sample collection and the 30-minute time window of cerebral hemodynamic data used to calculate corresponding values. Primary parameters assessed were the p-value of the fitted fixed slope effect and the adjusted R-squared of the fitted linear mixed effects model between MD parameter and cerebral hemodynamic parameter. MD parameters were log transformed prior to fitting due to significant non-normality. Optimal delay time was evaluated as the minimal slope p-value and maximal adjusted R-squared. The results of this sensitivity analysis are shown for LPR and glycerol MD biomarkers in "No DHCA" animals (DHCPB and SACP groups) and in animals with DHCA.

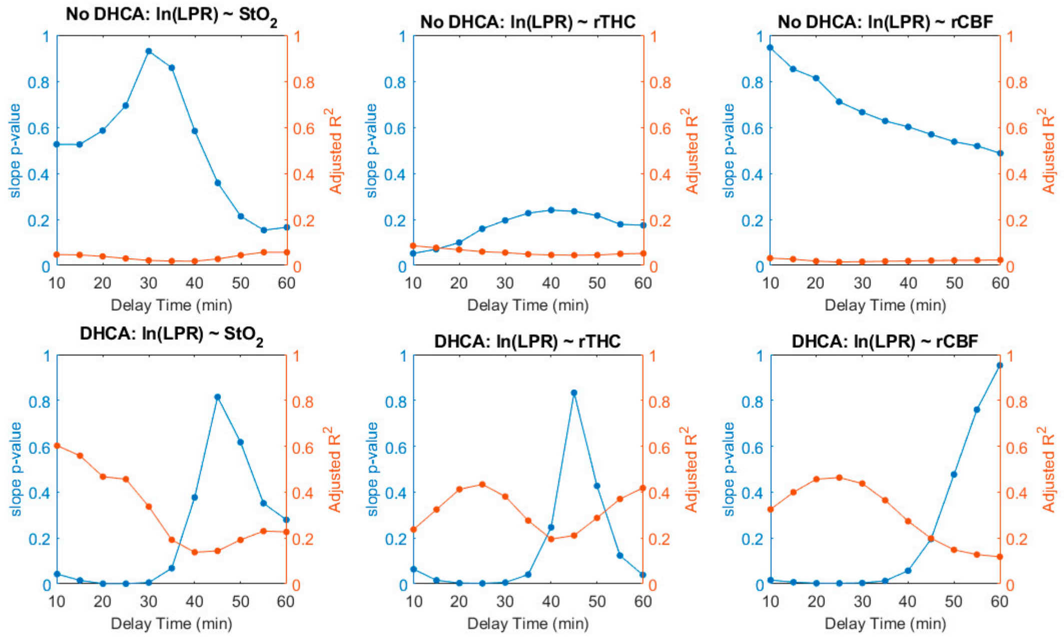

In "No DHCA" animals, there was poor linear model performance for the relationship between LPR and cerebral hemodynamics. At all delay times, no adjusted R-squared values exceeded 0.1. In contrast, in animals that underwent DHCA, a clear nadir in slope p-values (high significance) is observed at ~20 minutes. We thus selected a delay time of 20 minutes for the reported analysis to assess the causal relationship between changes in cerebral hemodynamics and resulting changes in cerebral metabolite.

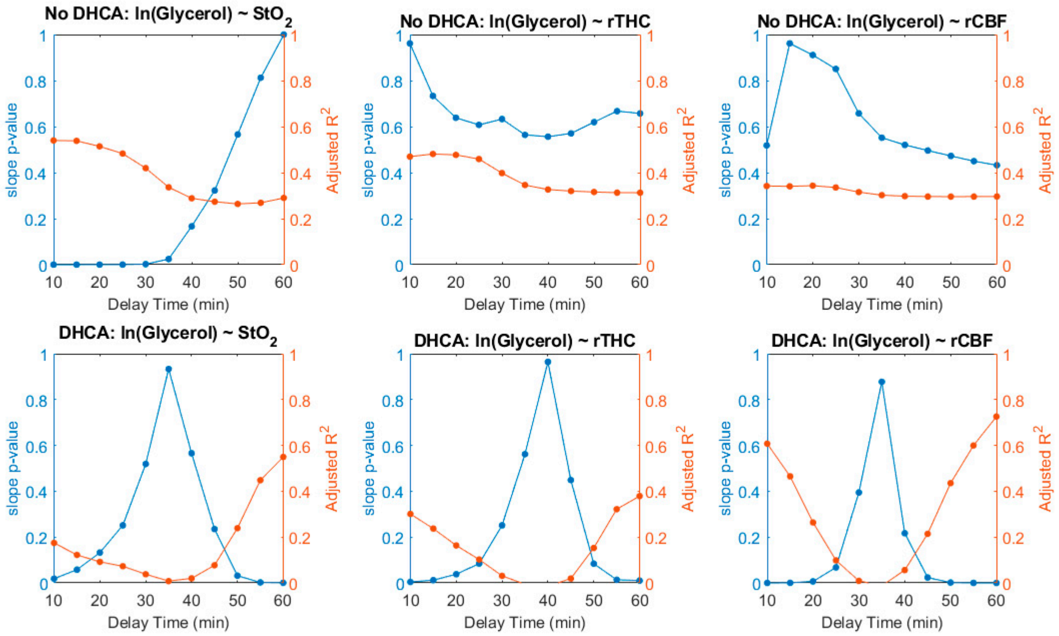

The sensitivity analysis with glycerol, however, yields different results. Examining the relationship of glycerol and cerebral hemodynamics, in "No DHCA" animals, linear model fits improved with adjusted R-squared values ranging between 0.3 to 0.6 for all parameters and delay times; maximal R-squared values were observed between 10-25 minutes. Significant slope p-values were observed in StO_2 from 10-35 minutes. In DHCA animals, optimal model performance (maximal adjust R-squared) is biphasic; significant slope p-values are observed at a delay of 10 minutes for StO_2 and 10-20 minutes for relative THC and CBF. Optimal performance is observed at a delay time of 60 minutes. Striking qualitative differences are observed in models at shorter delay times (positive correlation) versus longer delay times (negative correlation); these differences are highlighted in the main manuscript.

These results suggest variability in the timing and causal relationship between MD metabolites and cerebral hemodynamics. Variability is observed between metabolites; LPR appears to be modifiable by cerebral hemodynamics as optimal correlation is observed with positive delay. In addition to the transit time of the dialysate through the collection tube, the duration of delay may also be impacted by physiologic factors such as cerebral perfusion and oxygen availability; the lack of correlation in animals that did not experience cerebral ischemia offers additional evidence of this.

Sensitivity Analysis of Delay Time between Cerebral Microdialysis Sampling of Lactate-Pyruvate Ratio (LPR) and Glycerol

We examined the correlation of cerebral microdialysis (MD) metabolite values of LPR and glycerol in DHCA animals. We hypothesized that elevated LPR would be positively correlated with glycerol, a marker of cell membrane injury, as persistent hypoxic-ischemic metabolic stress is known to cause cell death. This analysis helped elucidate the timing of the associated rise in glycerol following elevation of LPR. MD samples were collected in 30 minute intervals. We explore the relationship of LPR versus glycerol in the same sample (Delay time = 0m), the subsequent sample (Delay time = 30m), and two samples following (Delay time = 60m). As the delay time is increased, number of available samples decreases due to the absence of subsequent sampling.

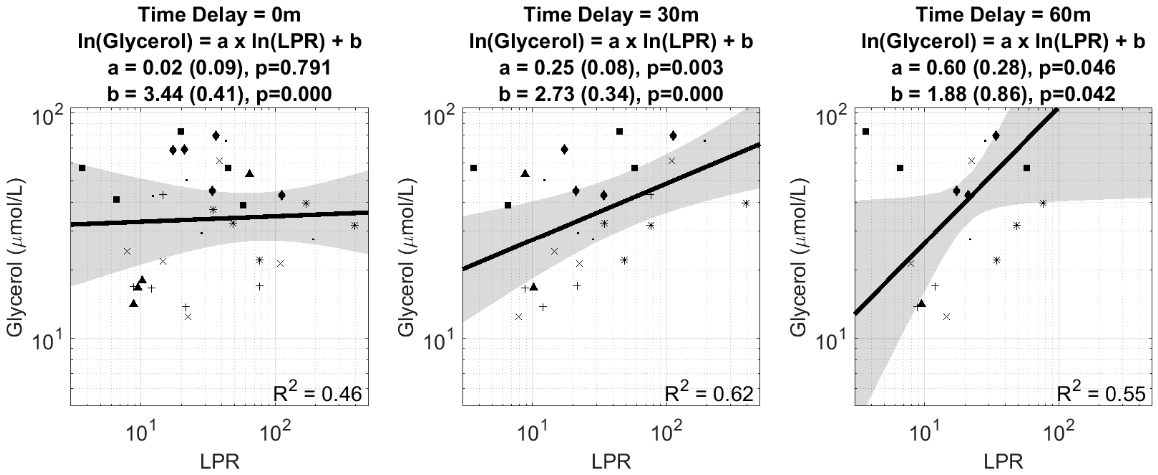

Significant correlation between LPR and Glycerol is detected at Delay times of 30m (slope p = 0.003) and 60m (slope p = 0.046), but not within the sample microdialysis sample (Delay time = 0m, slope p = 0.791). This suggests that in this model of transient hypoxic-ischemia, there is a 30–60 minute delay in elevation of glycerol, indicating cell death, following metabolic distress.
